# *In vivo* proton dosimetry using a MOSFET detector in an anthropomorphic phantom with tissue inhomogeneity

**DOI:** 10.1120/jacmp.v13i2.3699

**Published:** 2012-03-08

**Authors:** Ryosuke Kohno, Kenji Hotta, Kana Matsubara, Shie Nishioka, Taeko Matsuura, Mitsuhiko Kawashima

**Affiliations:** ^1^ National Cancer Centre Hospital East Kashiwa Chiba Japan; ^2^ National Cancer Centre Research Institute Chuo‐ku Tokyo Japan; ^3^ Research Fellow of the Japan Society for the Promotion of Science Tokyo Japan; ^4^ Graduate School of Human Health Sciences Tokyo Metropolitan University Arakawa‐ku Tokyo Japan; ^5^ Foundation for Promotion of Cancer Research Chuo‐ku Tokyo Japan; ^6^ Department of Medical Physics Hokkaido University Hospital Sapporo Hokkaido Japan

**Keywords:** MOSFET detector, *in vivo* proton dosimetry, anthropomorphic phantom, LET dependence, simplified Monte Carlo

## Abstract

When *in vivo* proton dosimetry is performed with a metal‐oxide semiconductor field‐effect transistor (MOSFET) detector, the response of the detector depends strongly on the linear energy transfer. The present study reports a practical method to correct the MOSFET response for linear energy transfer dependence by using a simplified Monte Carlo dose calculation method (SMC). A depth‐output curve for a mono‐energetic proton beam in polyethylene was measured with the MOSFET detector. This curve was used to calculate MOSFET output distributions with the SMC $\left({\text{SMC}}_{\text{MOSFET}}\right)$. The ${\text{SMC}}_{\text{MOSFET}}$ output value at an arbitrary point was compared with the value obtained by the conventional ${\text{SMC}}_{\text{PPIC}}$, which calculates proton dose distributions by using the depth‐dose curve determined by a parallel‐plate ionization chamber (PPIC). The ratio of the two values was used to calculate the correction factor of the MOSFET response at an arbitrary point. The dose obtained by the MOSFET detector was determined from the product of the correction factor and the MOSFET raw dose. When *in vivo* proton dosimetry was performed with the MOSFET detector in an anthropomorphic phantom, the corrected MOSFET doses agreed with the ${\text{SMC}}_{\text{PPIC}}$ results within the measurement error. To our knowledge, this is the first report of successful *in vivo* proton dosimetry with a MOSFET detector.

PACS number: 87.56.‐v

## I. INTRODUCTION

Comprehensive dose verifications are essential before radiation therapy with proton beams can be applied clinically. Because *in vivo* dosimetry can be used to identify major deviations in treatment delivery, its use during patient treatment may serve as the ultimate dose verification for patient quality assurance.

An *in vivo* dosimetry detector must be very small and easy to localize. Metal‐oxide semiconductor field‐effect transistor (MOSFET) detectors may be used to achieve this goal. The MOSFET detector has been used as a pinpoint dosimeter for the dose verification of photons^(^ ^1^ ^–^ ^5^ ^)^ and electrons.^(6)^ Although the MOSFET detector has been used for proton beam dosimetry,^(^ ^7^ ^,^ ^8^ ^)^ the relative response of the TN‐252‐RD MOSFET detector (Best Medical Canada, Ottawa, Canada) in the Bragg peak region was 26% lower than responses obtained with an ionization chamber,^(7)^ presumably because the MOSFET detector strongly depends on the linear energy transfer (LET) of the proton beam. These findings suggest that it may be difficult to measure the proton dose with a MOSFET detector.

To correct the MOSFET response to proton beams, Kohno et al.^(9)^ developed a simple dose‐weighted correction method. The MOSFET depth output was compared with the Bragg curve obtained by ionization chamber. A correction factor for the response of the MOSFET detector was calculated as a function of the proton penetration depth. Because the protons at any point may have a variety of energies due to multiple scattering effects, the correction factor at an arbitrary point can be calculated with the pencil beam dose calculation algorithm (PBA).^(^ ^10^ ^–^ ^12^ ^)^

It is possible to measure the absolute proton dose in a homogeneous phantom with the MOSFET detector by employing the correction method for LET dependence. However, the actual human body has internal structures with tissue inhomogeneity. During dose distribution under conditions of tissue inhomogeneity, protons pass through the irregular paths along various materials, such that there are complex hot and cold spots around the boundary of inhomogeneity.^(^ ^13^ ^–^ ^16^ ^)^

The proton dose has yet to be assessed by a simple dose‐weighted correction method under conditions of inhomogeneity. In particular, the PBA assumes that the central axis is a straight line, and determines the energy deposit and the lateral spread due to materials along the central axis; the PBA does not include the effects of lateral density inhomogeneity. Therefore, the correction method for LET effects strongly depends on the precision of the PBA calculation, which some authors have reported is less accurate under inhomogeneous conditions. As a result, the accuracies of these correction methods deteriorate in inhomogeneous media. For clinical use, further improvements to the dose calculation algorithm are desirable in situations involving tissues with significant inhomogeneity.

Here, we used a previously developed simplified Monte Carlo dose calculation method (SMC) to improve dose calculation accuracy under conditions of tissue inhomogeneity.^(^ ^13^ ^,^ ^14^ ^,^ ^16^ ^)^ The SMC results were compared to PBA calculations in the head and neck region of an anthropomorphic phantom. To correct the MOSFET response to proton beams, a highly precise correction method was developed with the SMC. Using the MOSFET detector with this correction and the PBA, we performed *in vivo* proton dosimetry in the head and neck region of an anthropomorphic phantom for therapeutic proton beams.

## II. MATERIALS AND METHODS

### A. Experimental apparatus

A commercially available MOSFET patient dose verification system (Best Medical Canada, Ottawa, Canada) was used. To reduce temperature dependence and nonlinear response at high‐dose levels, the TN‐252RD detector was constructed as a dual device containing two identical MOSFETs on the same chip.^(17)^ The sensitive field for each MOSFET was 0.2×0.2mm, and the oxide thickness was 0.25 mm. The detectors were 2×1.3×8mm in size, including the encapsulation. All measurements were performed with a high‐sensitivity bias voltage setting.

Measurements were performed by using the therapeutic proton beam line at our hospital. The beam line employs a dual‐ring double‐scattering method for proton therapy.^(18)^ The thickness of the first scatter and the shape of the second scatter were determined by the energy of the proton beams. The maximum size of the irradiation field provided by this system was 200 mm in diameter. For 190 MeV proton beam, daily testing was used to ensure that the proton range was within ±0.5mm.^(19)^

For accurate comparisons, the detector outputs were converted to dose values. Measurements were carried out in a PMMA dose calibration phantom for dose calibration.^(^ ^7^ ^,^ ^9^ ^)^ A calibrated 0.6 cc Farmer ionization chamber (FIC) type 30013 (PTW, Freiburg, Germany) and the MOSFET located within the phantom were placed along a line perpendicular to the beam axis. The proton energy and LET were 157 MeV and 0.5 keV/μm. Protons with this energy are protons of proximal region in Bragg curve, and the MOSFET detectors have no response changes due to LET dependence. To obtain the dose calibration factor for the MOSFET detector, the MOSFET and the FIC were exposed five times to 200 cGy. The dose calibration factor was determined from the average output. The raw dose for the MOSFET detector was obtained from the product of the MOSFET reading (mV) and the dose calibration factor.

**Figure 1:**
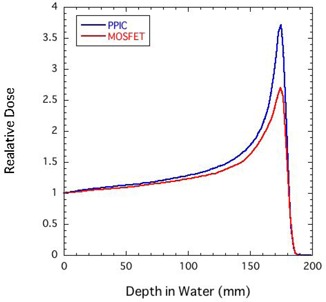
Comparison of Bragg curves obtained by using a parallel‐plate ionization chamber (PPIC) and MOSFET detectors for a 190 MeV proton beam.

The depth output curve in polyethylene slabs for mono‐energetic proton beams was measured by using the MOSFET detectors. The equivalent water thickness was calculated by multiplying the polyethylene thickness by 1.02. Figure 1 shows a comparison of Bragg curves obtained by using a parallel‐plate ionization chamber (PPIC) and MOSFET detectors for a 190 MeV proton beam. Both results were normalized to the response at a thickness of 0 mm. Compared with the result by the PPIC, the MOSFET response did not change in the shallow regions. On the other hand, the relative response of the MOSFET detector deteriorated with depth, and the MOSFET response at the Bragg peak was only 0.73. Thus, to measure proton dose with the MOSFET detector, the MOSFET response needs to be corrected.

**Figure 2:**
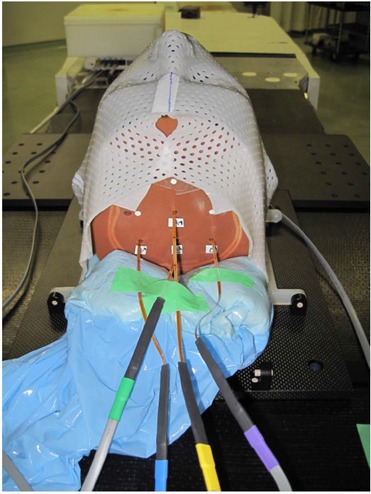
*In vivo* proton dosimetry using the MOSFET detector with an anthropomorphic phantom.

To evaluate the usefulness of the MOSFET detector as an *in vivo* dosimeter under more realistic conditions, *in vivo* proton dosimetry was performed by using the MOSFET detector with an anthropomorphic phantom (The Phantom Laboratory, Salem, CA) (Fig. 2). In particular, the head and neck region of the phantom reflects complex inhomogeneous tissues, with bone, soft tissue, various materials, and various shapes. The anthropomorphic phantom was immobilized with a mold and mask.

**Figure 3:**
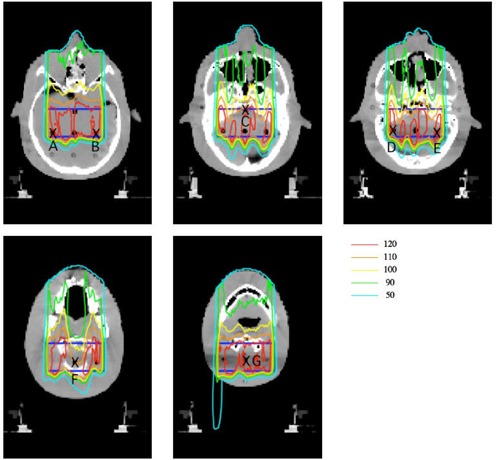
Axial images of the head and neck region in an anthropomorphic phantom, and isodose distributions calculated by the SMC. The Xs (black) represent the measurement points; the blue line is the target region.

Figure 3 shows a target region (blue line). A treatment plan was designed for the target in the head and neck region. We assumed the target shape is a rectangular solid, to make it easy to evaluate *in vivo* dosimetry using the MOSFET detector. A location of isocenter is a center of CT image. Gantry angle of 0° is arranged on the proton treatment planning system. For the target, a bolus and a patient collimator were designed by using the planning system. A 190 MeV proton beam, a ridge filter of 50 mm SOBP width and a range shifter of 7.5 mm thickness were selected. The Xs (black) in Fig. 3 represent the measurement points. Seven measurement points were selected to evaluate the proton dose formed by protons that pass through the inhomogeneities (e.g., jaw consisting of cortical bone, oral cavity consisting of air, brain stem, brain, etc.). In this experiment, the isocenter was exposed thrice to 100 cGy as a point prescription.

Axial isodose distributions calculated by the conventional SMC for the target in a head and neck region are shown in Fig. 3. In the target region, the dose distribution was not uniform, with large bumps and dips, such that there were steep gradients in the dose distributions of >5% per mm at some measurement points. Because there are complex hot and cold spots around the boundary of inhomogeneity, a precise dose calculation algorithm is desirable in situations involving tissues with significant inhomogeneity.

### B. Correction method of the MOSFET response

Because the correction method for LET effects is highly dependent on the precision of the PBA calculation, and as the PBA is limited to dose prediction for heterogeneous media, further improvements to the dose calculation algorithm are desirable for situations involving tissues with significant heterogeneity. We used the SMC^(^ ^13^ ^,^ ^14^ ^,^ ^16^ ^)^ to obtain a correction factor for the MOSFET response to proton beams under conditions of tissue heterogeneity.

The SMC method utilizes the measured depth‐dose distribution of a broad proton beam in water to calculate energy loss at a given depth. The energy loss of a proton in a segment of material was calculated with the water equivalent model. The SMC method begins by tracking individual protons at the entrance to the range compensator. The initial beam parameters were provided by the effective source model^(10)^ on the basis of the measurements. The effective source model provides the standard deviation of the initial angular distribution at any point on the entrance plane. The proton fluence distribution was determined on the basis of the measured lateral dose distribution. A calculation grid size of 2 pixels was used for the CT image. The pixel size of the CT image had an area of 0.586mm×0.586 mm and a thickness of 3 mm. Each particle was characterized in terms of its position, direction, and residual range. The trajectory of each particle was tracked by assuming multiple Coulomb scattering. The scattered projection angles were expressed as normal random numbers.

${\text{SMC}}_{\text{MOSFET}}$ used the measured depth output curve of Fig. 1 to calculate the MOSFET output distributions. The MOSFET output values at an arbitrary point obtained by ${\text{SMC}}_{\text{MOSFET}}$ were compared with the dose values obtained by the conventional ${\text{SMC}}_{\text{PPIC}}$, which uses the depth‐dose curve determined using the PPIC. When this calculation was used for Fig. 3 (as an example), the estimated mean statistical error of the calculated dose in the target volume region was within 2% rms.

The correction factor for the MOSFET detector ${\text{CF}}_{\text{SMC}}(x,y,z)$ can be calculated at an arbitrary point, by using:

(1) $$CF_{SMC}(x,y,z)=\frac{\sum_{i=1}^{n}SMC_{PPIC}^{i}(x,y,z)}{\sum_{i=1}^{n}SMC_{MOSFET}^{i}(x,y,z)}$$

Where *i* is the ith proton and *n* is the total number of protons. The dose measured by the MOSFET detector at (x,y,z) is given by the product of ${\text{CF}}_{\text{SMC}}(x,y,z)$ and the raw MOSFET dose without correction for the MOSFET response.

A simple dose‐weighted correction method with the PBA has been already developed.^(7)^ For mono‐energy proton beams and a spread‐out Bragg peak, this method uses the correction factor as a function of proton penetration depth. For each irradiation condition (e.g., proton energy, spread‐out Bragg peak), approximation functions of the proton penetration depth must be measured and determined, respectively. Therefore, it is hard to apply the simple dose‐weighted correction method to clinical use with various irradiation conditions.

In order to improve the simple dose‐weighted correction method with the PBA, we developed a new correction method with the PBA that would be easier to use and which does not need the above preliminary work. Additionally, since this method calculates directly a MOSFET correction factor, this method would be more accurate than the simple dose‐weighted correction method, which uses approximation functions such as the function of the proton penetration depth. Results with this method were compared with the results corrected with the SMC to evaluate the correction method of MOSFET response with the SMC.

In general, the dose ${\text{PBA}}_{\text{PPIC}}(x,y,z;(x_{0},y_{0}))$ delivered by a single pencil beam at an entrance position $\left(x_{0},y_{0}\right)$ is given by:

(2) $$\begin{array}{l} PBA_{PPIC}(x,y,z;(x_{0},y_{0}))=\phi (x_{0},y_{0})DD(z;(x_{0},y_{0}))\\ \;\times \frac{1}{2\pi \sigma {\left(z\right)}^{2}}\exp\left(-\frac{{\left(x_{0}-x\right)}^{2}-{\left(y_{0}-y\right)}^{2}}{2\sigma {\left(z\right)}^{2}}\right) \end{array}$$

where $\phi (x_{0},y_{0})$ is the intensity profile of the broad beam, $\text{DD}(z;(x_{0},y_{0}))$ is the depth‐dose distribution of the broad beam measured by the PPIC, and σ(*z*) is the proton spread due to multiple scattering effects in the bolus and polyethylene slabs and the configuration of the beam line at *z*. We defined the MOSFET output $\left({\text{PBA}}_{\text{MOSFET}}(x,y,z;(x_{0},y_{0})\right)$ delivered by a single pencil beam as in Eq. (2):

(3) $$\begin{array}{l} PBA_{MOSFET}(x,y,z;(x_{0},y_{0}))=\phi (x_{0},y_{0})DO(z;(x_{0},y_{0}))\\ \;\times \frac{1}{2\pi \sigma {\left(z\right)}^{2}}\exp\left(-\frac{{\left(x_{0}-x\right)}^{2}-{\left(y_{0}-y\right)}^{2}}{2\sigma {\left(z\right)}^{2}}\right) \end{array}$$

where $\text{DO}(z;(x_{0},y_{0}))$ is the depth output distribution of the broad beam measured by the MOSFET detector.

Finally, the correction factor for the MOSFET response ${\text{CF}}_{\text{PBA}}(x,y,z)$ is given by:

(4) $$CF_{PBA}(x,y,z)=\frac{\sum_{i=1}^{n}PBA_{PPIC}(x,y,z;(x_{i},y_{i}))}{\sum_{i=1}^{n}PBA_{MOSFET}(x,y,z;(x_{i},y_{i}))}$$

where *i* is the ith pencil beam, *n* is the total number of pencil beams, and $(x_{i}$,$y_{i})$ is the position of a generated pencil beam. The dose measured by the MOSFET detector at (x,y,z) is given by the product of ${\text{CF}}_{\text{PBA}}(x,y,z)$ and the raw MOSFET dose.

## III. RESULTS & DISCUSSION

**Figure 4:**
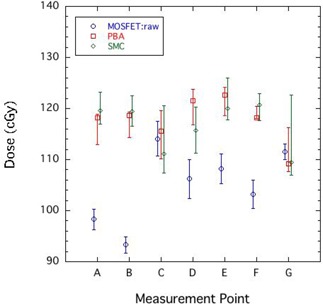
Comparison of doses obtained by the uncorrected MOSFET detectors (MOSFET:raw), PBA, and SMC at measurement points A–G.

Figure 4 compares the doses obtained by the uncorrected MOSFET detectors (MOSFET:raw), PBA, and SMC at measurement points A–G. The error bars of MOSFET:raw represent the reproducibility of the MOSFET measurements, and is the standard deviation. The error bars of PBA estimate difference between dose at the evaluation point and maximum/minimum dose in a cavity size of 5 mm in diameter due to the MOSFET setup uncertainty. On the other hand, the error bars of SMC include difference between dose at the evaluation point and maximum/ minimum dose in a cavity size of 5 mm in diameter due to the MOSFET setup uncertainty, and a statistical error of 2%.

At each measurement point in the target region, the dose varied from 110 to 120 cGy. It is obvious that dose distributions in the target region were not simply uniform. The maximum difference between the PBA and the SMC was 5.0%. As expected, there was a large difference for dose prediction precision in tissue heterogeneity between both dose calculations. The MOSFET:raw values at measurement points A, B, D, E, and F markedly underestimated the SMC results by about 15%. Figure 4 reveals obvious deteriorations of the MOSFET measurements at these points in the spread out Bragg peak within the target region. As a result, these results require correction for MOSFET response.

In contrast, the measurement values at C and G were similar to those obtained by the SMC. As shown in Fig. 3, dose distributions at C and G formed a large bump and dip structure, even in the target region. From the error bars of the SMC calculation in Fig. 4, we can observe that measurement points C and G had steep gradient dose distributions of >5% per mm. In other words, the measurement uncertainties at C and G tended to be larger than those at other points.

**Figure 5:**
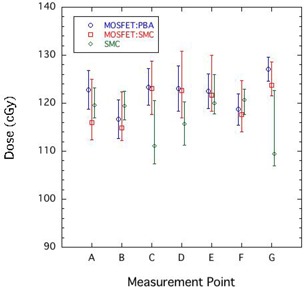
Comparison of doses obtained by the corrected MOSFET detectors with the PBA (MOSFET:PBA), the corrected MOSFET detectors with the SMC (MOSFET:SMC), and SMC at measurement points A–G.

Figure 5 compares the doses obtained by the corrected MOSFET detectors with the PBA (MOSFET:PBA), the corrected MOSFET detectors with the SMC (MOSFET:SMC), and SMC at measurement points A–G. The error bars of MOSFET:PBA and MOSFET:SMC also estimate errors of the correction factor in each calculations due to the MOSFET setup uncertainty. Namely, they include dose error caused by the difference between correction factor at the evaluation point and maximum/minimum correction factor in a cavity size of 5 mm in diameter.

There was some difference from 5.9% to 0.2% in the MOSFET:PBA and the MOSFET:SMC. These differences depended on the precision of the dose calculation algorithm in inhomogeneities. Including the results at C and G, the results of MOSFET:SMC agreed with the SMC results within the error bars at all points. This result indicates that we could correct the MOSFET response accurately, even in a situation with large and complex inhomogeneities.

However, the average magnitude of the error bars for MOSFET:SMC was about ±4.6%, which indicates that large measurement errors were unavoidable in *in vivo* dosimetry. In other words, it is difficult to measure accurate point doses, such as at measurement points C and G. To improve the accuracy of *in vivo* dosimetry, an image‐guided system is needed that can accurately monitor the position of the MOSFET detector.

## IV. CONCLUSIONS

To measure the proton dose in tissue inhomogeneities with a MOSFET detector, we developed a highly precise method using the SMC to correct the MOSFET response to proton beams. Additionally, we improved the simple dose‐weighted correction method to be easy to use. In our experimental evaluation with an anthropomorphic phantom, there were some differences between the MOSFET:PBA corrected by the PBA and the MOSFET:SMC corrected by the SMC. The MOSFET:SMC agreed well with results calculated by the SMC within the measurement error. To our knowledge, this study is the first to report successful *in vivo* proton dosimetry with a MOSFET detector under conditions with large tissue inhomogeneities.

## ACKNOWLEDGMENTS

We thank Dr. A. Hallil, Best Medical Canada, for his support in the form of materials and special prototypes. We are grateful to Kazutomo Matsumura and Ryuichi Oota, SHI Accelerator Service Ltd., for their experimental support. This work was supported in part by a Grant‐in‐Aid for Young Scientists (B) (No. 21791236) from the Japan Society for Promotion of Science (JSPS).

## Supporting information

Supplementary Material

Supplementary Material
