# Supplementary figures and images for: In vivo proton dosimetry using a MOSFET detector in an anthropomorphic phantom with tissue inhomogeneity
Source: J Appl Clin Med Phys. 2012 Mar 8;13(2):159–67. doi: 10.1120/jacmp.v13i2.3699 (PMC5716407; doi:10.1120/jacmp.v13i2.3699)

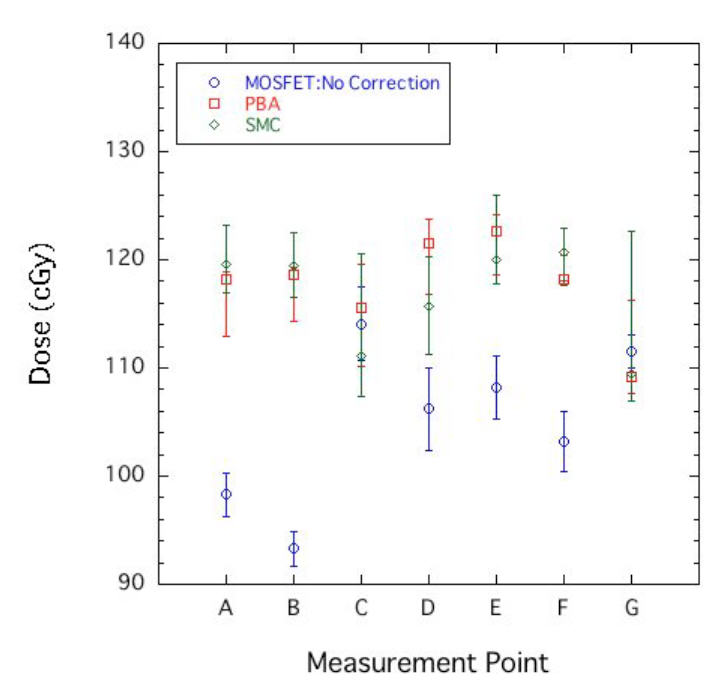

Supplement: Supplementary file 1 — Supplementary Material [file ACM2-13-159-s001.jpg]

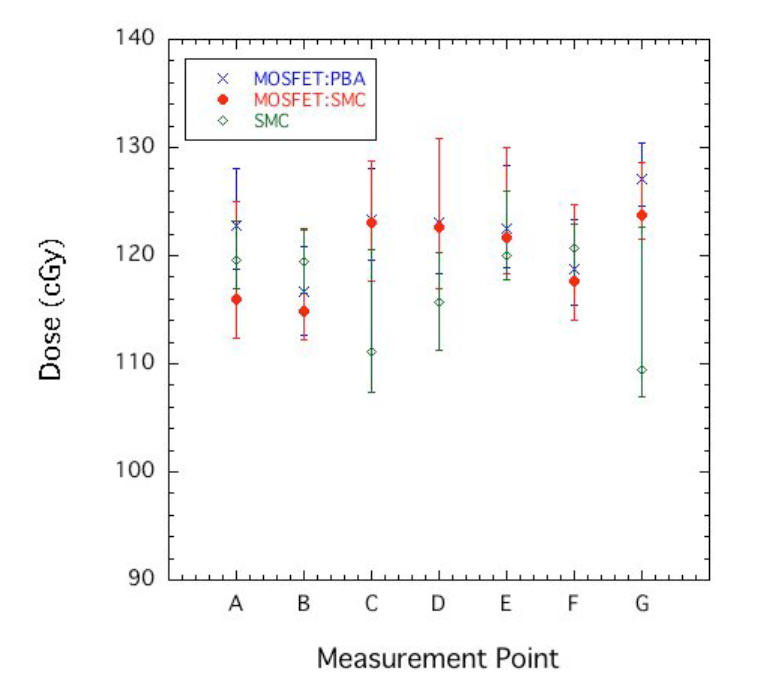

Supplement: Supplementary file 2 — Supplementary Material [file ACM2-13-159-s002.jpg]
